# Complete Genome Sequence of a Novel GV.2 Sapovirus Strain, NGY-1, Detected from a Suspected Foodborne Gastroenteritis Outbreak

**DOI:** 10.1128/genomeA.01553-14

**Published:** 2015-02-12

**Authors:** Shinichiro Shibata, Tsuyoshi Sekizuka, Akari Kodaira, Makoto Kuroda, Kei Haga, Yen Hai Doan, Reiko Takai-Todaka, Kazuhiko Katayama, Takaji Wakita, Tomoichiro Oka, Hiroyuki Hirata

**Affiliations:** a Microbiology Department, Nagoya City Public Health Research Institute, Aichi, Japan; b Pathogen Genomics Center, National Institute of Infectious Diseases, Tokyo, Japan; c Department of Virology II, National Institute of Infectious Diseases, Tokyo, Japan; d Nagoya City Public Health Research Institute, Aichi, Japan

## Abstract

Sapoviruses, members of the family *Caliciviridae*, are genetically highly diverse. We report here the first complete genome sequence of a genogroup V genotype 2 sapovirus strain, NGY-1, detected from fecal samples of a suspected foodborne gastroenteritis outbreak, determined using a metagenomic sequencing approach.

## GENOME ANNOUNCEMENT

Sapovirus (SaV) is a pathogen causing gastroenteritis in humans. Based on their complete capsid-encoding region nucleotide sequence, SaV strains detected from humans have recently subdivided into multiple genogroups and genotypes (i.e., GI.1-7, GII.1-7, GIV.1, and GV.1) (1). We recently identified a sapovirus strain, Hu/SaV/GV/NGY-1/2012/JP (SaV NGY-1), from fecal samples of a suspected foodborne gastroenteritis outbreak that occurred in Aichi Prefecture, Japan, on 3 April 2012, using a metagenomic sequencing approach. SaV NGY-1 was classified as belonging to a new genotype in the genogroup V (GV.2) based on statistically defined cutoff pairwise distance values (2). In this study, we report the first complete genome sequence of the GV.2 sapovirus strain.

Viral RNA was extracted from fecal suspensions using the High Pure viral RNA kit (Roche). A 200-bp fragment library was constructed using the NEBNext Ultra RNA library prep kit for Illumina version 1.2 (New England BioLabs) and then purified using Agencourt AMPure XP magnetic beads (Beckman Coulter). The quality of the purified library was assessed on an MCE-202 MultiNA bioanalyzer (Shimadzu Corporation) and the concentration determined on a Qubit 2.0 fluorimeter using the Qubit HS DNA assay (Invitrogen). A 151-cycle paired-end read sequencing was carried out on a MiSeq sequencer (Illumina) using the MiSeq reagent kit version 2 (300 cycles). FASTQ formatted sequence data were analyzed using the CLC Genomics Workbench software version 7.5.1 (CLC bio), and the nearly full-length SaV-NGY-1 strain genome sequence was first obtained by *de novo* assembly. The 5′-terminal nucleotide sequence of the SaV-NGY1 strain genome was further determined by a DNA-DNA ligation method, as described elsewhere (3). The purified PCR products corresponding to the 5′-end region of the genomes were sequenced directly using the BigDye Terminator cycle sequence kit version 3.1 and the capillary sequencer 3500xL (Applied Biosystems). The full-length genome sequence of the SaV NGY-1 strain was assembled by the Sequencher program version 4.10.1 (GeneCodes) and analyzed by Genetyx-Mac software version 16.0.4 (Genetyx Corporation).

The genome of the SaV GV.2 NGY-1 strain consists of 7,521 nucleotides (nt), excluding the poly(A) tail. The genome was predicted to contain two major open reading frames (ORFs) in nucleotide positions 15 to 6920 (ORF1) and 6917 to 7420 (ORF2). The predicted 5′ and 3′ untranslated regions (UTRs) are 14 nt and 101 nt long, respectively.

The 5′ end of the genome starts with GTG, similar to that of other sapoviruses, and the first 18 nt of the 5′ genome sequence of the GV.2 NGY-1 strain are identical to the corresponding sequences of human origin GV.1 SaV strains Ehime475/Japan (GenBank accession no. DQ366344) and NongKhai-24/Thailand (GenBank accession no. AY646856).

The SaV NGY-1-like strains, based on the partial capsid sequences (nucleotide identity, 92%; within ~300 nt), have also been detected in the stool of a child with gastroenteritis (PR 4900/2009/Italy [GenBank accession no. JQ303049]) (4), and from river water (Llobregat River/Site5_2/Mar2009/ES [GenBank accession no. AB559909]) in spain (5), and wastewater (isolates SaV/Water-5 and SaV/Water-6 [GenBank accession no. DQ915088 and DQ915092, respectively]) in Japan (6). These results suggest the ubiquitous circulation of GV.2-like SaV strains in different countries.

### Nucleotide sequence accession number. 

The genome sequence of SaV NGY-1 strain has been deposited in GenBank under the accession no. AB775659.
